# Sensor-based phenotyping of above-ground plant-pathogen interactions

**DOI:** 10.1186/s13007-022-00853-7

**Published:** 2022-03-21

**Authors:** Florian Tanner, Sebastian Tonn, Jos de Wit, Guido Van den Ackerveken, Bettina Berger, Darren Plett

**Affiliations:** 1 Australian Plant Phenomics Facility, School of Agriculture, Food and Wine, University of Adelaide, Urrbrae, SA Australia; 2 Department of Biology, Plant-Microbe Interactions, Utrecht University, 3584CH Utrecht, The Netherlands; 3 Department of Imaging Physics, Delft University of Technology, Lorentzweg 1, 2628 CJ Delft, The Netherlands

**Keywords:** Plant disease, Phenotyping, Imaging sensors, Plant-pathogen interactions, Biotic stress, Signs and symptoms

## Abstract

Plant pathogens cause yield losses in crops worldwide. Breeding for improved disease resistance and management by precision agriculture are two approaches to limit such yield losses. Both rely on detecting and quantifying signs and symptoms of plant disease. To achieve this, the field of plant phenotyping makes use of non-invasive sensor technology. Compared to invasive methods, this can offer improved throughput and allow for repeated measurements on living plants. Abiotic stress responses and yield components have been successfully measured with phenotyping technologies, whereas phenotyping methods for biotic stresses are less developed, despite the relevance of plant disease in crop production. The interactions between plants and pathogens can lead to a variety of signs (when the pathogen itself can be detected) and diverse symptoms (detectable responses of the plant). Here, we review the strengths and weaknesses of a broad range of sensor technologies that are being used for sensing of signs and symptoms on plant shoots, including monochrome, RGB, hyperspectral, fluorescence, chlorophyll fluorescence and thermal sensors, as well as Raman spectroscopy, X-ray computed tomography, and optical coherence tomography. We argue that choosing and combining appropriate sensors for each plant-pathosystem and measuring with sufficient spatial resolution can enable specific and accurate measurements of above-ground signs and symptoms of plant disease.

## Background

Worldwide yield losses in major crops due to pathogens and pests are estimated to be 17–30% [1]. In this review, we will focus on plant pathogens, i.e., organisms or biotic agents that can cause disease [2] and not on pests such as insects and nematodes. Plant pathogens belong to various taxa including viroids, viruses, phytoplasmas, bacteria, oomycetes and fungi [3]. When a pathogen interacts with a plant, structural, physical, and biochemical changes can occur in both the plant and the pathogen. Depending on plant genotype, pathogen strain, and environmental conditions, the outcome of plant-pathogen interactions (PPI) may be disease, a physiological disturbance of the plant [3–5].

Disease resistance breeding and precision agriculture are key strategies to reduce yield losses due to plant disease in a sustainable way. Both rely on detection, identification and quantification of plant disease on various scales. In disease resistance breeding and pre-breeding, PPI are examined at the cell, tissue, whole plant and field plot level. Zooming in to the cell or tissue level can uncover the distinct mechanisms that determine plant resistance or susceptibility, and precise quantification of disease or resistance levels in whole plants or field plots aids the selection of the best genotypes. In precision agriculture, early and precise disease detection in the field enables efficient crop protection, e.g. by targeted pesticide application or eradication of diseased plants. The challenge to detect and quantify plant disease in an unbiased and precise way initiated the field of plant disease phenotyping [6–8].

In general, “plant phenotyping” describes the study of the manifestation of a genotype under specific environmental conditions [9]. In the context of PPI the phenotype consists of changes that can be described as contrasting indications of disease: signs and symptoms. Whereas these terms were originally used for changes that are visible to the human eye, here we will use them also for changes that can be detected by non-invasive sensors.

**Fig. 1:**
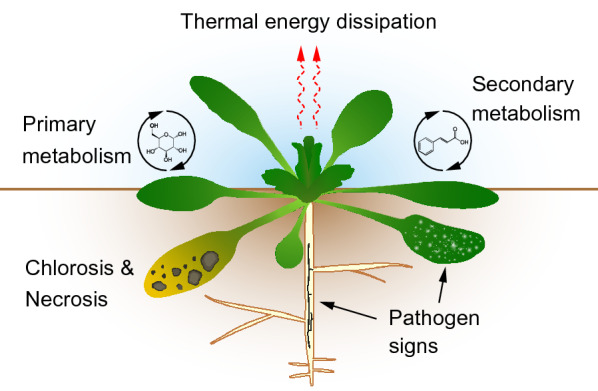
Signs and symptoms of plant-pathogen interactions. Depicted skeletal formulas are glucose, representing primary metabolism, and cinnamic acid, representing secondary metabolism

Following the American Phytopathological Society (APS) Illustrated Glossary of Plant Pathology, a “symptom” is an indication of disease by reaction of the host [2]. These plant reactions include changes to pigmentation (e.g. necrosis, chlorosis), primary and secondary metabolism, and thermal energy dissipation (Fig. 1). A “sign” is an indication of disease from direct observation of a pathogen or its parts (e.g. sporulation, formation of fruiting bodies, mycelium, bacterial ooze) [2].

While the signs and symptoms are specific for each plant-pathosystem and influenced by environmental conditions, we classify them by their shared characteristics for this review. In practice, signs and symptoms most often do not appear in isolation but occur simultaneously. For example, chlorosis, necrosis, and sporulation may successively co-occur in the same area of an infected leaf.

Phenotyping of PPI can be addressed with invasive methods. For example, colonization of a plant leaf by a pathogen can be detected, classified and quantified by quantitative polymerase chain reaction (qPCR) or for bacterial pathogens by measuring colony-forming units in a homogenate. Such invasive methods can be precise and objective. However, they are necessarily destructive and limited in speed and scalability, limitations that can be overcome by non-invasive sensors.

**Fig. 2:**
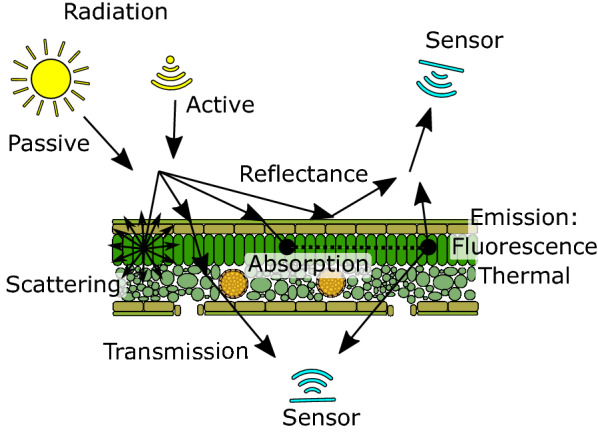
Physical paths of electromagnetic radiation in biological samples and their detection using non-invasive sensors. Passive (ambient light) or active radiation can be used to illuminate or excite the sample. Radiation can be reflected, transmitted, scattered, absorbed and re-emitted by the sample to varying degrees. The characteristic radiation can then be measured with sensors positioned on the side of the source of illumination or on the opposite side of the sample

Non-invasive sensing offers the possibility of time-course measurements, higher throughput and lower costs [8, 10, 11]. The classic approach for non-invasive phenotyping is visual inspection by humans. This can yield precise and accurate estimates if raters are well trained and appropriate scales are used. However, visual estimates are prone to subjectivity, offer limited speed and scalability, are often qualitative rather than truly quantitative, and are innately limited to the visible spectrum of light [12]. Sensor-based non-invasive phenotyping has the potential to increase throughput and precision, and can detect disease signs and symptoms that are invisible to the human eye [7]. Essentially, non-invasive sensors capture the changes in interactions between electromagnetic radiation and matter (Fig. 2).

The most established sensors for non-invasively measuring PPI are red–green–blue (RGB), hyperspectral, thermal, and fluorescence sensors (Table 1). Less often, monochrome sensors, Raman spectroscopy, and tomographic sensors have been used [6, 13–16].

**Table 1:** Summary of sensors that have been used for phenotyping PPI

Sensor, technology	Imaging/non-imaging	Active/passive	Effect measured	Excitation/illumination wavelengths	Measured wavelengths
Monochrome	Imaging	Mainly active	Reflectance	Variable	Variable
RGB	Imaging	Mainly active, passive at large scale	Reflectance	Variable, usually visible spectrum	Range: ~ 400–700 nm R: ~ 600 nm G: ~ 530 nm B: ~ 460 nm
Hyperspectral	Both	Mainly active, passive at large scale	Reflectance, transmission	Variable	400–2500 nm
Thermal	Mainly imaging	Passive	Emission	NA	8–15 µm
Chlorophyll fluorescence (kinetics)	Imaging/non-imaging	Active	Emission	400–700 nm	~ 650–800 nm
Fluorescence	Imaging/non-imaging	Active	Emission	Mostly 300–400 nm	Mainly 400–700 nm
Raman spectroscopy	Non-imaging	Active	Inelastic scattering of photons (Raman scattering)	Variable, often 785–830 nm [17]	Raman bands, 400–2133 cm^−1^ [18]
Optical coherence tomography	Imaging	Active	Reflectance of coherent light	800–1000 nm or 1200–1400 nm	800–1000 nm or 1200–1400 nm
X-ray computed tomography	Imaging	Active	Attenuation, phase shift	~ 0.01–0.1 nm	Visible light using scintillator

By focusing here on the biological aspects of disease phenotyping, namely the signs and symptoms, we highlight the sensor-based technologies that are most suitable for specific plant-pathosystems. We group the signs and symptoms into five generalised categories (Fig. 1 and Table 2) to illustrate the common biological processes that underlie sensor-detected signals, to highlight similarities between different plant-pathosystems and to thereby point out possibilities to transfer and adapt phenotyping solutions (Table 3).

**Table 2:** Applications of sensor-based phenotyping of PPI

Sign/symptom	Plant	Pathogen	Sensor / Vector	Scale	Reference
Pathogen signs	*Arabidopsis thaliana*, *Hordeum vulgare*	*Fusarium graminearum* expressing GFP	Zeiss Stemi-C dissecting microscope with a 470 nm excitation filter and 535 nm emission filter	Whole plants (At), detached spikes (Hv)	[26]
	*Beta vulgaris* L.	*Cercospora beticola*	Hyperspectral microscope (PFD V10E), motorized stage	Individual lesions	[22]
	Cereals	*Blumeria graminis*	Monochrome CCD sensor, 4 channels captured	‘Macrobot’, robotic arm	[21]
	*Nicotiana benthamiana*	*Phytophthora infestans* expressing RFP	Monochrome camera with filter wheel and excitation lights (PathoScreen imaging system, Phenovation, the Netherlands)	Detached leaves	[28]
	*Nicotiana tabacum*	*Tobacco mosaic virus* expressing GFP	RGB camera under UV illumination (Blak-Ray Model B 100AP)	Detached leaves	[27]
	*Phaseolus vulgaris*	*Pseudomonas syringae *pv.* phaseolicola* expressing *lux-*eYFP operon	nightOWL LB 983 in vivo imaging system (Berthold Technologies, Germany), confocal laser scanning microscope Zeiss LSM 880	Detached leaves	[34]
	*Triticum aestivum* L.	*Fusarium graminearum*	X-ray CT, Biomedical Imaging and Therapy beamline (BMIT‐BM, 05B1‐1)	Spikelet	[114]
	*Vitis* spp.	*Erysiphe necator*	RGB (Nikon D850), automated motorized stage	Individual leaf discs, automated imaging	[20]
Primary metabolism	*Arabidopsis thaliana*	*Pseudomonas syrinae*	CF Imager (Technologica Ltd., UK), NPQ, fPSII, Fv/Fm	Individual plants	[149]
	*Cucumis melo*	*Podosphaera xanthii*	Open FluorCam 700 MF (Photon System Instruments), NPQ, fPSII	Individual leaves	[54]
	*Hordeum vulgare*	*Blumeria graminis* f. sp.* hordei*	Chlorophyll Fluorometer IMAGING-PAM M-series (Walz, Germany)	Individual leaves	[150]
	*Lactuca sativa*	*Bremia lactucae*	Open FluorCam 700 MF (Photon System Instruments), Fv/Fm	Leaf discs	[151]
	*Nicotiana benthamiana*	Pepper mild mottle virus	FluorCam (Photon System Instruments), NPQ, fPSII	Individual leaves	[152]
	*Olea europaea*	*Xylella fastidiosa*	Micro-hyperspectral imager (VNIR model, Headwall Photonics, USA), 400 – 885 nm, from aircraft 500 m above ground	Orchards	[48]
	*Oryza sativa*	*Rhizoctonia solani*	Non-imaging NeoSpectra micro handheld spectrometer (SiWare Systems, Canada), 1348–2551 nm	Individual leaf spots (non-imaging)	[61]
	*Solanum tuberosum*	*Phytophthora infestans*, *Alternaria solani*	Non-imaging field spectrometer SVC HR-1024i (350–2500 nm) (Spectra Vista Corporation, USA)	Individual leaf spots (non-imaging)	[153]
Secondary metabolism	*Beta vulgaris*	*Cercospora beticola*	SWIR spectral camera, 970–2500 nm (HySpex SWIR-320 m-e line camera, Norsk Elektro Optikk A/S, Norway)	Detached leaves	[154]
	*Citrus sinensis*, *Citrus paradisi*	*Candidatus* Liberibacter spp*.*	Non-imaging handheld Raman spectrometer (Resolve spectrometer equipped with 831-nm laser source, Agilent, USA)	Detached leaves	[86, 87, 90]
	*Hordeum vulgare*	*Blumeria graminis f.sp. hordei*	UV line scanner, 250–500 nm (Headwall Photonics)	Detached leaves	[81]
	*Nicotiana benthamiana*	Pepper mild mottle virus	Excitation with xenon-lamp + BP 340/75, imaging with CCD camera + BP 440/20 and BP 520/20	Individual leaves	[152]
	*Triticum aestivum*	*Puccinia triticina*, *Blumeria graminis* f. sp.* tritici*	Non-imaging fiber-optic fluorescence spectrometer (IOM GmbH, Germany) combined with 337 nm pulsed N2 laser	Individual leaf spots (non-imaging)	[73]
	*Vitis vinifera*	*Plasmopara viticola*	Macroscope (AZ100 multizoom, Nikon), ex. BP 340/26 and em. LP 371	Leaf parts	[69]
Necrosis and chlorosis	*Arabidopsis thaliana*	*Pseudomonas syringae*	RGB (Nikon D5200 DSLR)	Seedlings growing in well-plates	[117]
	*Arabidopsis thaliana*	*Sclerotinia sclerotiorum*	RGB (USB camera, full HD 1080p) controlled by Raspberry Pi 3 Model B motherboards	Detached leaves	[115]
	*Beta vulgaris*	*Cercospora beticola*	RGB (camera Baumer HXG-40), multispectral camera (6 bands of 10 nm between 450 and 850 nm, AIRPHEN)	Phenomobile 1, 50 distance to canopy top (RGB), hexacaopter (multispectral)	[119]
	*Triticum aestivum*	*Mycosphaerella graminicola*	RGB (flatbed scanner)	Detached leaves collected from field trial	[23]
	*Zea mays*	*Setosphaeria turcica*	RGB	Drone, 6 m above ground	[118, 120]
Thermal energy dissipation	*Cucumis sativus* L.	*Fusarium oxysporum *f. sp.* cucumerinum*	FLIR SC620	Individual leaves	[123]
	*Ipomoea batatas* L.	Sweet potato feathery mottle virus (SPFMV), Sweet potato chlorotic stunt virus (SPCSV)	Top-view thermal camera (FLIR A615), PlantScreen conveyor system, NaPPI, Helsinki	Whole plant	[132]
	*Nicotiana tabacum* L.	Tobacco mosaic virus (TMV)	Infrared imager (Agema THV900LW), Cartesian positioning system in imaging chamber	Leaves	[131]
	*Olea europaea* L.	*Verticillium dahliae*	Temperature sensor (Apogee IRR-P), Fixed 1 m above canopy	Single tree canopy	[129]
	*Olea europaea* L.	*Verticillium dahliae*	Broad-band thermal camera (FLIR SC655) on crewed aircraft	3000 ha, spatial resolution = 62 cm	[130]
Structural changes	*Capsicum annuum*	*Stemphylium lycopersici*	Laboratory-OCT system, 4096-pixel line scan camera (spl4096-140 km, Basler)	Single leaves	[134]
	*Malus domestica*	*Marssonina coronaria*	Backpack-based OCT system, 2048-pixel line scan camera (spL2048-140 km, Basler, Germany)	Single leaves	[133]
	*Solanum tuberosum*	*Streptomyces scabies*	Medical X-ray CT scanner (Toshiba Xvision high-resolution CT scanner)	Single plants	[135]
	*Triticum aestivum*	*Fusarium graminearum*	Synchrotron-based phase contrast X-ray imaging with the Biomedical Imaging and Therapy beamline (BMIT‐BM, 05B1‐1) at the Canadian Light Source	Single excised wheat spikes	[114]

**Table 3:** Suitability of sensors for phenotyping PPI

		RGB	Hyperspectral	Thermal	Fluorescence	Chlorophyll fluorescence (kinetics)	Raman spectroscopy	OCT	X-ray CT
Pathogen signs	Controlled	+ +	+	−	+	−	−	+	+
	Field	+ +	+	−	−	−	−	+	−
Primary metabolism	Controlled	−	+	−	−	+ +	+	−	−
	Field	−	+	−	−	+ +	+	−	−
Secondary metabolism	Controlled	−	+	−	+	−	+	−	−
	Field	−	+	−	+	−	+	−	−
Necrosis and chlorosis	Controlled	+ +	+ +	−	+	+	−	+	+
	Field	+ +	+	−	+	+	−	+	−
Thermal energy dissipation	Controlled	−	−	+ +	−	−	−	−	−
	Field	−	−	+	−	−	−	−	−

“Not used/unsuitable” (−), “Preliminary” ( +) and “Widely used” (+ +)

## Signs, symptoms and sensors

### Pathogen signs

After successful infection, plant pathogens propagate on or inside their host plant, either by rapid replication (e.g. bacteria, viruses) or mycelial growth (fungi, oomycetes) [4]. Quantifying the pathogen, based on signs like spores or mycelium, is a direct measure of plant resistance, defined in a strict sense as the ability of the host to restrict pathogen growth [19]. When a pathogen grows on the surface of the plant, sensors that directly capture the optical changes caused by its physical presence can be used for non-invasive measurement (Table 3).

Powdery mildews are surface-colonizing pathogens representing a variety of obligate biotrophic fungi that can cause disease on various host plants. Growing on the surface, these fungi only penetrate epidermal cells and use haustoria to acquire nutrients. RGB imaging was successfully applied to quantify pathogen signs both for grapevine powdery mildew (*Erysiphe necator*) and cereal powdery mildew infection (*Blumeria graminis* spp.) [20, 21]. In both studies, mycelial growth on detached leaf pieces was imaged in automated phenotyping systems. For grapevine powdery mildew, this system included a movable stage and a DSLR camera [20]. For cereal powdery mildew, a monochrome charge-coupled device (CCD) camera was combined with narrow-bandwidth illumination and a robotic arm system. The best correlation to visual estimates of infected area was achieved with a simple segmentation algorithm that uses the minimum of the three RGB values [21]. While the throughput of imaging can be easily increased compared to visual scoring for both cereal powdery mildew and grapevine powdery mildew, the preparation of leaf samples remains a bottleneck for these plant-pathosystems.

Unlike powdery mildews, many other filamentous pathogens like fungi and oomycetes form signs on the surface of the host only at the end of the disease cycle, in the form of spore bearing structures. Sporulation of *Cercospora beticola*, a polycyclic necrotrophic ascomycete fungus, occurs on the leaves of infected sugar beet in the area of the necrotic lesions. Hyperspectral microscopy was used to show that sporulation is correlated to an overall decrease in reflectance in the area of lesions in the spectral range of 400–900 nm [22]. However, the advantage of hyperspectral images over RGB images in this study is unclear since the proposed trait is the difference of the integral of reflectance over the entire spectral range of the camera. It would be interesting to know whether the difference in reflectivity over a narrower wavelength range (e.g. one of the RGB channels) could match or improve the quantification of fungal sporulation.

An RGB flatbed scanner was used to assess the interaction between a panel of 335 wheat cultivars and *Mycosphaerella graminicola* (Septoria tritici blotch) [23]. Leaves were collected from a field trial with natural infection and scanned. From the scans the density, size and melanisation of pycnidia (signs) as well as lesion size (a symptom) were measured. A Genome-Wide Association Study using the phenotypic data identified 26 chromosome intervals associated with Septoria tritici blotch resistance. Sixteen of these loci overlapped with intervals that had already been identified based on visual assessment in previous studies, while ten had not been described before, demonstrating the potential power of quantitative phenotyping [23]. This example also illustrates the benefit of a high resolution, e.g. being able to discriminate single pycnidia to determine density and size, thus overcoming the weakness of low spatial resolution of many field phenotyping methods.

Transgenic pathogen strains that express detectable markers such as fluorescent proteins or bioluminescence conferring enzymes are another alternative to track and quantify pathogen growth directly [24, 25]. Fluorescent proteins have mainly been used to study the infection process in vivo at the cell level using epi-fluorescence or fluorescence confocal laser scanning microscopy. But also at the level of whole plants or seedlings, pathogens expressing fluorescent proteins, including bacteria, fungi, oomycetes and viruses have been used to track, image and quantify infection and colonization [26–31]. Plant autofluorescence and low fluorescent protein signal intensity can hinder imaging at larger scales. Bioluminescence, so far mainly applied in bacteria, generates a light signal without prior excitation, therefore avoiding plant autofluorescence. But the low signal intensity requires imaging with sensitive cameras and extended exposure time in the dark (up to several minutes) [32, 33]. A recent study generated bioluminescent and fluorescent *Pseudomonas syringae* pv. *phaseolicola* [34]. The bioluminescence enabled detection of the bacteria at the leaf scale in a dedicated imaging chamber. Identified colonized plant parts could then be sampled and further investigated under the fluorescence microscope making use of the expressed fluorescent proteins. Since enzymes producing bioluminescent compounds have also been identified in fungi, such a luminescence based approach might also be feasible to facilitate quantification and examination of fungal infection at the macro- and microscopic level [35]. But all these approaches are restricted by the requirement for both a protocol for genetic transformation of the pathogen of interest and for facilities authorized to carry out experiments with transgenic plant pathogens.

### Symptom: changes in primary metabolism

In plant-pathogen interactions, the plant primary metabolism is influenced both by manipulation of the pathogen and the immune response of the plant itself. Pathogen infection may modify source-sink relations in the plant or impair photosynthesis, while plant immune responses require additional resources from the pool of primary metabolites [36–38]. Together this may lead to detectable symptoms based on photosynthetic performance or altered accumulation and allocation of primary metabolites.

Photosynthetic performance can be probed by analyzing chlorophyll *a* fluorescence and the kinetics of chlorophyll *a* fluorescence (Chl-F) [39, 40]. According to the model of photosystem II (PSII) absorbed light energy can take three different paths: (i) drive photosynthesis (photochemical quenching); (ii) dissipate as heat (non-photochemical quenching); (iii) re-emit as fluorescence [39, 41].

Measuring the kinetics of Chl-F, the changes of Chl-F under different light conditions, e.g. before and after a saturating light pulse, allows separation of these components and calculation of diverse parameters that yield information about photosystem II (PSII) photochemistry, electron flux, and CO_2_ assimilation [39, 40]. Commonly used parameters are the maximum quantum efficiency of PSII photochemistry (F_v_/F_m_), the operating efficiency of PSII photochemistry (ɸPSII, F_q_’/F_m_’ or ∆F/F_m_), the level of photochemical quenching of PSII (qP or F_q_’/F_v_’) or the level of non-photochemical quenching (NPQ) which estimates the rate constant for heat loss from PSII [40].

With commercially available Chl-F kinetics imaging systems these parameters can be mapped onto imaged leaves or whole plants, enabling identification of spatial heterogeneity that may be linked to localized pathogen infection [42]. Many of the Chl-F parameters (e.g. F_v_/F_m_, qP, NPQ) are measured on dark-adapted plants and commonly require light sources in close proximity to the plant to provide e.g. a saturating pulse to measure maximum fluorescence (F_m_). Therefore, most Chl-F imaging systems are designed for growth chambers or greenhouses where LED panels can provide even illumination and plants can easily be dark adapted [42]. However, there are also field phenotyping systems that include Chl-F imaging with active illumination [43]. Dark adaption in the field can be achieved by imaging at night or before dawn. But because the sensor needs to be close to the plants, Chl-F imaging with active illumination in the field is limited to ground-based phenotyping platforms which offer limited throughput compared to uncrewed aerial vehicles (UAV).

Analysis of spectral reflectance under sunlight does not require active illumination and is therefore an alternative for probing photosynthesis that is compatible with aerial vectors like UAVs or aircrafts. Spectral reflectance data can be used to build predictive models for photosynthetic parameters like maximum carboxylation rate of Rubisco or to estimate sun-induced chlorophyll fluorescence [44–47]. For example, sun-induced chlorophyll fluorescence, determined from spectral images taken from an aircraft, has successfully been used to estimate disease severity of olive trees infected with the bacterium *Xylella fastidiosa* [48]*.* But these approaches are technically challenging, both in data acquisition and data analysis, and interpretation is difficult because the relationship of reflectance, canopy geometrical structure, leaf physiology and variation in solar radiation is not fully understood [47, 49, 50]. So far, these challenges limit applications, despite the potential especially for large scale remote sensing of plant stress.

Chlorophyll fluorescence imaging systems with active illumination on the other hand have been used in numerous studies to monitor the effect of pathogen infection on plants [42]. A common response, in many cases prior to visual changes, is the decrease of ɸPSII resulting from a decreased PSII electron transport as well as an increased heat dissipation rate (NPQ). This has also been observed for infections of many biotrophic pathogens like powdery mildew of wheat and barley (*B. graminis*), powdery mildew of cucurbits (*Podosphaera xanthii*), downy mildew of lettuce (*Bremia lactucae*) and downy mildew of grapevine (*Plasmopara viticola*) [51–54]. These biotrophic pathogens often induce visible symptoms only at late infection stages, thus Chl-F imaging may be particularly useful to visualize and quantify early colonization.

A general drawback of Chl-F imaging is the lack of specificity as photosynthesis and Chl-F are influenced by many biotic and abiotic stress factors alike [55]. This could be partially overcome by taking into account the differences in spatial patterns of Chl-F changes. Patterns induced by localized pathogen infection might be distinguishable from patterns induced by abiotic stresses that affect the whole plant.

While Chl-F provides information about the current productivity of the plant, it does not allow for quantification of the actual concentration of primary metabolites. Changes in accumulation and allocation of sugars, starch, amino acids or proteins can, in principle, also be estimated directly via reflectance spectroscopy or imaging spectroscopy in the visible (VIS, 400–700 nm), near-infrared (NIR, 700–1000 nm) and shortwave infrared (SWIR, 1000–2500 nm) range [56–58]. Such approaches are based on combining non-invasive spectroscopic measurements with biochemical analysis of the same tissue to build predictive models. For example, Ely et al*.* [56] developed spectra-trait models for leaf starch, glucose, and protein content based on reflectance spectroscopy (500–2400 nm) and biochemical analysis of leaves of eight crop species. However, the usefulness of such models for linking spectral features to metabolic changes during PPI still requires validation.

So far, studies only indicate that reflectance spectroscopy may sense specific changes in primary metabolism during PPI. Gold et al. [59] collected reflectance spectra (400–2400 nm) with a portable non-imaging contact spectrometer from potato leaves at different infection stages of *Phytophthora infestans* or *Alternaria solani*. Using spectra-trait models they estimated pathogen-induced changes in leaf sugar, starch and nitrogen concentration and found increased sugar concentration during the biotrophic, necrotrophic and sporulation phase of *P. infestans*. But these estimates were not validated by chemical analysis and the applied spectra-trait models were originally developed on data from forests and grasslands [60]. Although the study shows clear differences in reflectance spectra between tissue infected with the different pathogens and tissue at different infection stages, the interpretation of these spectral differences remains unclear.

A similar study in rice with healthy and sheath blight (*Rhizoctonia solani*) affected plants found that differential spectral regions could be linked to absorption features of starch, cellulose and protein content, although they were not chemically validated [57, 61].

Both of these studies used non-imaging spectrometers and found spectral features in the SWIR range to be important for detection of diseased plant tissue. Therefore, imaging SWIR sensors might be particularly useful to not only measure changes to primary metabolites but also to provide spatial information on these changes.

### Symptom: changes in secondary metabolism

Plant secondary metabolites (PSM) are a large group of structurally and functionally diverse metabolites that are, as opposed to primary metabolites, considered non-essential for primary functions like photosynthesis, growth and reproduction [62, 63]. Those PSM that are involved in plant immunity are commonly classified into two groups, phytoanticipins and phytoalexins [64]. While phytoanticipins are constitutively produced and stored in plant tissue, phytoalexins are synthesized in response to a pathogen. Members of both groups show in vitro antimicrobial and insect-deterrent activity [65].

Independent of their function, PSM can be informative markers for preformed resistance (phytoanticipins), or a symptom of the infection progress, and magnitude or quality of the plant immune response (phytoalexins). Due to their specific absorption and, in some cases, fluorescence spectra they may be detected non-invasively, e.g. by reflectance and fluorescence spectroscopy or imaging [57, 66].

In grapevine, infection with the downy mildew pathogen *Plasmopara viticola* induces the accumulation of stilbenes, a group of phenolic compounds [67]. Pure stilbenes emit a violet-blue fluorescence around 400 nm when excited with UV light (335 nm) [68]. A fluorescence signal with a similar spectrum was imaged in downy mildew infected grapevine leaves at the cell level using confocal laser scanning microscopy, as well as at the tissue level using epifluorescence macroscopy [68–70]. Mass spectrometry imaging revealed co-localization of stilbenes with the violet-blue fluorescence signal, suggesting that stilbenes are indeed the source or at least contribute to the observed fluorescence [70]. Since stilbene synthase expression has also been linked to downy mildew resistance, the violet-blue fluorescence may not only enable detection and quantification of downy mildew infection but also allow for the assessment of differences in plant defense responses [71].

Blue (around 440 nm) and green (around 520 nm) fluorescence upon UV excitation has also been described in *Nicotiana benthamiana* infected with Pepper mild mottle virus, likely due to accumulation of the phenolic compound chlorogenic acid, and in wheat infected with leaf rust (*Puccinia triticina*) or powdery mildew (*Blumeria graminis *f. sp.* Tritici*) [72, 73].

These examples indicate that changes in UV-excited blue and green fluorescence, induced by certain pathogens, is a conserved response across plant species. In fact, it has been described as a general conserved stress response, also to abiotic stresses including drought, nutrient deficiencies and increased UV irradiation [74–77]. Responsible fluorophores in most cases are likely stress-induced soluble and cell wall bound phenolic compounds that fluoresce in the blue-green spectrum [78]. For example, the fluorescent stilbenes accumulate in grapevine leaves also in response to prolonged UV-C irradiation [70]. Consequently, distinction between infections of different pathogens, or between biotic and abiotic stress, might not be possible. This is a critical limitation for phenotyping in field trials, where various stresses can occur simultaneously.

Plant secondary metabolite content of leaves may also be estimated via reflectance spectroscopy [79, 80]. Spectral indices or models for leaf traits like phenolic content have been mostly developed and validated for remote sensing in landscape ecology studies [60, 79]. For example, Kokaly and Skidmore [80] proposed that an absorption feature around 1660 nm is related to content of phenolic compounds in different plant species and showed that in fresh tea leaves (*Camellia sinensis*), this absorption feature correlates with total phenolic compound content.

Only a few studies have combined spectral measurements with biochemical analysis of diseased plants. This is required to link spectral features to physiological processes during PPI. Brugger et al. [81] explored spectral imaging in the UV range (250–400 nm), which is particularly interesting because many plant secondary metabolites involved in stress responses feature absorption maxima in that range. They found in barley infected with powdery mildew (*Blumeria graminis *f. sp. *hordei)* that changes in flavonoid content during the first 5 days of infection correlated with reflectance intensity around wavelengths that match flavonoid absorption spectra. But adverse interaction of the sensor with the required UV light source restrains interpretation of these results. Another study combined spectral imaging in the SWIR range (970–2500 nm) with untargeted metabolic fingerprinting of three different sugar beet genotypes infected with *Cercospora beticola* [82]*.* Although there were correlations between several secondary metabolites and spectral data, it remains unclear if this correlation is due to direct contribution of these metabolites to the reflectance spectrum. Combining imaging spectroscopy with mass spectrometry imaging could help to provide direct links between specific metabolite groups and spectral features.

Raman spectroscopy is another technology to measure changes in plant secondary metabolism. After excitation of the sample with a laser it measures the inelastic scattering of photons (also called Raman scattering), which can provide both qualitative and quantitative information about the chemical composition of the sample [15, 83]. Raman scattering can be collected with portable non-imaging handheld Raman spectrometers [84, 85] and this approach has been applied to detect viral, bacterial and fungal infections in plants [85–89]. For example, a handheld Raman spectrometer was used to detect *C. liberibacter* spp. infection in citrus trees. Using orthogonal partial least squares discriminant analysis, grapefruit leaves were classified into healthy, infected, and nutrient-deficient categories with 98% accuracy in the training set, but the authors did not validate the classification accuracy in a test set [86, 87]. Infection was associated with increased intensity of the Raman band assigned to lignin and phenolic compounds. Correspondingly, a follow-up study found increased *p*-coumaric acid content in infected leaves, a phenolic compound and lignin precursor whose Raman spectrum matches the disease associated bands [90]. Similar disease associated bands, likely corresponding to phenolic compounds, have also been described for virus infection in wheat, tomato and rose [15, 88, 91]. These studies used non-imaging Raman spectrometers, which only provide point measurements and do not yield any spatial information. But Raman spectroscopy can also be combined with digital imaging so that Raman spectra are recorded for each pixel [92]. This has been explored as a tool for quality and safety inspection in food, pharmaceutical, and biomedical sectors and, for example, to detect watermelon seeds infected by the bacteria *Acidovorax citrulli* [93, 94]. So far, the lengthy image acquisition has restricted the throughput and therefore applications of Raman imaging, but Lee et al. [94] report a relatively fast system that requires 250 s to image an area of five by twenty cm with a spatial resolution of 250 by 1024 pixels. Such systems may already be useful for certain phenotyping challenges, but further reducing the acquisition time would widen the range of possible applications.

### Symptom: necrosis and chlorosis

Pathogen-induced chlorosis and necrosis are prominent symptoms of plant disease as they are visually evident and very common. Chlorosis results from changes in pigmentation, mainly the degradation of chlorophyll, and necrosis from the death of cells and tissue. Both may occur locally in lesions, with chlorosis often preceding or surrounding necrotic lesions. Viruses can cause chlorosis in diverse patterns that are often reflected in their name (e.g. “chlorosis”, “mottle”, “mosaic”, “streak”, “vein clearing”, “yellowing”) [95].

Both chlorosis and necrosis can be induced by specific pathogen-produced metabolites or proteins, e.g. the chlorosis inducing coronatine from *Pseudomonas syringae* or the necrosis and ethylene-inducing peptide 1 (Nep1)-like proteins from *Botrytis cinerea* [96–101]. Particularly pathogens with a necrotrophic life style produce toxins that kill plant cells, by disrupting the plant cell membrane directly or by generating membrane damaging reactive oxygen species [102, 103]. In interactions with viruses and biotrophic pathogens like downy mildews, chlorosis is often induced in later stages of the infection, and chlorophyll degradation appears to be regulated by the same plant genes that control regular leaf senescence [104–106]. The area of chlorotic or necrotic tissue can serve as a good proxy to estimate spread of the pathogen, as well as the impact on yield, depending on plant developmental stage, type of the pathogen and stage of the infection.

While chlorotic tissue has reduced chlorophyll content, necrotic tissue lacks all pigments characteristic for healthy plant tissue. This results in changes of absorption and reflectance in the visible spectrum, evident as color ranging from yellow (chlorosis) to shades of brown to black (necrosis). Besides the lack of pigments, necrotic tissue also differs from healthy tissue in water content and three-dimensional structure due to the collapse of cells. A lower ratio of cell surface to intercellular air space due to a collapse of e.g. the spongy mesophyll leads to reduced reflectance of NIR radiation [107]. Changes in water content in necrotic tissue also impacts SWIR reflectance due to several water absorption peaks in the SWIR range [108, 109]. Consequently, sensors that detect reflectance in the VIS–NIR-SWIR range are useful to quantify chlorosis and necrosis. The contrast between healthy leaf tissue and lesions lacking chlorophyll may be enhanced by imaging the red steady-state chlorophyll fluorescence [110, 111]. The onset of cell death can also be visualised by imaging the increased chlorophyll fluorescence that results from the disassembly of the chloroplast thylakoid membrane in tissue undergoing cell death [112]. Additionally, the changes in plant internal tissue structure that precede and are associated with necrosis and tissue damage can be measured with different tomography methods, e.g. optical coherence tomography (OCT) or X-Ray computed tomography (CT) [113, 114].

In controlled experiments, particularly on samples that are easy to image such as detached leaves, RGB imaging is an established method to track and quantify chlorosis and necrosis. Barbacci et al. [115] combined a detached leaf assay with a setup for automated RGB image acquisition and analysis to quantify necrotic lesion development on Arabidopsis inoculated with *S. sclerotiorum*. This enabled measurement of latency period and lesion doubling time (LDT) at high resolution (measurement every 10 min over 36 h) and of large sample sizes (120–270 leaves per imaging unit). Whereas latency period varied mostly between different *S. sclerotiorum* isolates, LDT was mainly determined by the plant genotype and independent of the inoculated isolate. Using the differences in LDT as a robust indicator of quantitative resistance led to the identification of the nucleotide-binding site leucine-rich repeat gene *LAZ5* as a negative regulator of quantitative resistance to *S. sclerotiorum.* On a similar scale, RGB imaging enabled quantification of chlorosis induced by *P. syringae* in Arabidopsis seedlings growing in 48-well plates [116, 117]. The assay was tested in a genome-wide association study to efficiently distinguish between presence and absence of effector-triggered immunity and confirmed the loci of known resistance genes.

In field experiments, necrosis in maize infected by *Setosphaeria turcica* (Northern corn leaf blight) or sugar beet infected by *C. beticola* (Cercospora leaf spot) has been assessed by sensors [118, 119]. Wiesner-Hanks et al. [118, 120] acquired images with a RGB camera mounted on a UAV flying 6 m above a maize field trial. The necrotic lesions captured in these images were manually annotated and used to train a CNN. Combining this CNN with a conditional random field method allowed automated segmentation of the aerial images to identify and quantify lesion area.

In a sugar beet field trial, Jay et al. [119] tested both RGB imaging from a ground-based vehicle and spectral imaging (six bands between 450 and 850 nm) from a UAV to assess Cercospora leaf spot severity. They determined the necrotic spot density from RGB images and green fraction from both RGB and spectral images. The image data was compared to visual severity scores given by an expert on a 1–9 scale. Spot density gave a better prediction for low (less severe) visual scores and green area was a better predictor for high visual scores. Consequently, combining these two features as input for a neural network enabled a good prediction of the visual disease scores. Because only the ground-based RGB sensor enabled measuring both of these features, it outperformed the aerial spectral sensor.

### Symptom: thermal energy dissipation

Plant-pathogen interactions can result in a change of tissue temperature by affecting energy balance terms such as transpiration or light absorption [121]. These induced changes often precede other symptoms and are characterized by complex spatial and temporal dynamics. This makes thermal energy dissipation an interesting candidate trait for early detection of disease. PPI can cause an increase in tissue temperature by inducing stomatal closure and vascular occlusion. Conversely, damage to cells and deregulation of stomatal opening can lead to decreased tissue temperature through uncontrolled transpiration [122, 123]. Photosynthetic performance has an influence on plant temperature as well because part of the absorbed light energy, if not emitted as fluorescence or converted in photochemistry, is dissipated as heat in the process of non-photochemical quenching [39].

Temperature can be remotely measured in the thermal infrared spectrum (TIR, 8000–15,000 nm) with radiometric sensors [124]. This technology has long been applied to measure abiotic stresses, particularly drought stress which is associated with an increased canopy temperature [125, 126]. Multiple plant-pathosystems have also been studied with thermal sensors [127].

Vascular pathogens that grow within the xylem of host plants can cause occlusion of the vascular system due to both their own growth and the plant responses to the pathogens. This can lead to symptoms similar to drought stress, as the hydraulic conductivity is inhibited by the occlusion [128]. In a field experiment, the crown temperature of olive trees under natural infection with *Verticillium dahliae*, a vascular fungal pathogen, was measured with infrared temperature sensors (Apogee IRR-P) mounted 1 m above the trees. The temperature was positively correlated with Verticillium wilt disease severity levels across multiple sites [129]. The same authors validated and vastly increased the throughput of their methods by mounting a broad-band thermal camera (FLIR SC655) on a crewed aircraft and flying it over 3000 ha of olive orchards [130].

The measurement of plant thermal energy dissipation is not easy to measure in the field as environmental effects and the influence of other stresses decrease specificity of the measurements. Therefore, many studies of plant thermal energy dissipation are performed in controlled environments.

An abscisic-acid-induced stomatal closure in leaves of cucumber infected with the vascular pathogen *Fusarium oxysporum* f. sp. *Cucumerinum* could be detected by an increase in temperature with a FLIR SC620 digital infrared camera in a controlled environment [123]. The maximum temperature of leaves of infected plants was reached nine days after infection. The authors also observed a fast decrease of temperature ten days after infection and attributed it to uncontrolled water loss due to cell damage. Eleven days after infection, leaf temperature rose again which was attributed to dehydration of the leaves.

Virus-plant interactions can also influence tissue temperature. Tobacco infected with tobacco mosaic virus shows a fast increase in leaf temperature at the initial infection site preceding a hypersensitive response. This increase in temperature is due to stomatal closure which is induced by salicylic acid accumulation. The same areas of the leaves later appear as necrotic lesions [131]. Changes in leaf temperature were also observed in sweet potato upon infection with two different viruses, Sweet potato feathery mottle virus (SPFMV) and Sweet potato chlorotic stunt virus (SPCSV) [132]. In this controlled environment experiment, leaf temperature was measured with a top-view thermal camera (FLIR A615) and differed between healthy plants, plants infected with SPFMV, and plants co-infected with both viruses. Higher temperatures were associated with higher disease severity scores [132].

### Sensing structural changes

Preliminary studies using tomographic sensors for phenotyping PPI have been performed in various plant-pathosystems. As these sensors measure spatially resolved attenuation, refractive index variation and scattering strength inside tissue, they can be useful to non-invasively study internal structural changes which can allow for early detection and potentially identification of disease [113, 114, 133, 134]. Besides measuring internal plant and pathogen structure, X-ray CT scanning also provides the option to measure through substrate and was used to study changes in root morphology of potato affected by *Streptomyces scabies* [135].

Synchrotron‐based phase contrast X-ray CT was used to measure differences in tissue degradation in wheat caused by *Fusarium graminearum* [114]. A known resistance mechanism to this fungus is the inhibition of fungal colonization from spikelet to spikelet inside the rachis internode. Traditional histological studies to quantify this type of resistance require destructive sampling. The tissue degradation leads to increased tissue porosity, which makes it possible to sense the pathogen spread by changes in X-ray attenuation.

Leaves of apple trees infected with *Marssonina coronaria* were measured with a custom-built backpack-based OCT sensor in the field [133]. Disease progression causes an enlarged gap between epidermis and palisade parenchyma that could be sensed by a reduction in backscattering. The presence of infection was confirmed using Loop-mediated isothermal amplification, a nucleic-acid based technique [133]. Authors from the same group applied similar techniques in leaves of *Capsicum annuum* infected with *Stemphylium lycopersici* [134].

These studies show the potential of tomographic sensors for early detection of disease as well as phenotyping of below ground structures.

## Discussion

### Phenotyping plant-pathogen interactions in the field is limited by specificity, canopy structure, and environmental conditions

Signs and symptoms are easier to measure in a controlled environment where plant material can be accessed from multiple angles at close proximity and under optimal illumination. Yet field phenotyping is a requirement for most disease resistance breeding programs and precision agriculture.

Traits identified in controlled environments at small scales such as lesion size or sporulation, may, in some cases, be transferrable to the field [7, 136, 137]. For example, Northern corn leaf blight causes large and obvious lesions on maize plants, a symptom that was measured on RGB images taken from a UAV at 6 m altitude [118, 120]. This way, the relative area of necrotic maize tissue could be measured with high throughput.

However, it is technically more demanding to measure signs and symptoms under field conditions. Multiple biotic and abiotic stresses can affect plants simultaneously and lead to a loss of specificity. For example, chlorosis may be caused both by a pathogen or abiotic stress. In a canopy, plants or plant parts can shade each other from the sensors, especially in tall growing crops where lower plant parts are covered. Applying sensors from a distance, e.g. when mounted on UAVs, can result in a lack of spatial resolution. Also, natural radiation influences sensor-based measurements and can confound the measurement of signs and symptoms.

The effect of natural radiation can be controlled either by numerical correction during the data analysis, or by shading during the data acquisition, but both approaches are difficult due to the spatial and temporal variation of natural radiation. The technical limitations involving spatial resolution and natural radiation could be overcome partly by ground-based phenotyping platforms that carry sensors close to the canopy and offer a compromise between throughput and accuracy. For example, ground-based RGB imaging outperformed aerial RGB imaging for assessing Cercospora leaf spot in a sugar beet field trial [119]. The ground-based platform offered higher spatial resolution and artificial illumination which enabled measuring necrotic spot size and density, traits that could not be measured with the aerial platform. The specificity of measurements could potentially be further improved by sensor fusion, the integration of data from multiple sources [138, 139]. A combination of functional plant traits derived from hyperspectral imaging (400–885 nm) and a thermal imaging sensor mounted on an aircraft enabled early detection of *Xylella fastidiosa*, a xylem-bound bacterial pathogen of olive trees [48].

These examples illustrate that it is feasible to increase throughput while maintaining accuracy also under field conditions. But it is critical that the challenges of specificity, canopy architecture, spatial resolution and natural radiation are considered and addressed with new solutions like improved sensor and vector technology or sensor fusion.

### Non-invasive phenotyping of below-ground plant-pathogen interactions remains a challenge

Phenotyping of PPI below ground is still in an early stage of development as in-soil non-invasive phenotyping is difficult. Using invasive sampling and RGB imaging, changes of morphological root characteristics could be detected on soybean infected with *Fusarium* species and on alfalfa affected by Phymatotrichopsis root rot [140, 141]. Tomographic sensors are an option to measure PPI on roots growing in substrate in pots non-invasively [135]. With new X-ray CT scanners that are installed in automated phenotyping facilities, this method may become more accessible [142–144].

### Sensor data helps to understand plant-pathogen interactions in more detail

Sensor-based phenotyping is commonly deployed to substitute traditional visual disease scores, for example to rank a genotype on a spectrum from resistant to susceptible in comparison with other genotypes [12]. Yet sensor-based phenotyping can capture the sum of processes that underlie PPI in more detail than what is reflected in traditional disease severity scores. To understand which specific PPI related changes influence the detected signals, sensor data needs to be linked with in situ measurements, particularly in the case of advanced sensors. This has been shown successfully in two studies that linked disease induced accumulation of phenolic compounds to specific Raman bands in Raman spectroscopy [90] and flavonoids to UV absorption features in spectral imaging [81]. Establishing and confirming such links will allow to non-invasively measure diverse aspects of PPI simultaneously and to transfer those findings between pathosystems and environments.

### Advanced sensors expand the range of perceivable signs and symptoms but require complementary technologies

Implementation of sensor technology for phenotyping of PPI allows for measurement of a wide range of signs and symptoms (Table 2). Plant metabolites may be detected with spectroscopic methods, and internal plant and pathogen structures can be detected with tomographic methods [15, 18]. Fluorescence imaging, possibly with tagged pathogens, and chlorophyll fluorescence imaging are other promising approaches for measuring pathogen growth and photosynthetic parameters. To build useful phenotyping systems, any improvements and innovation in sensor technology need to be matched with appropriate facilities, vector technology, data management and data analysis methods. In those fields, constant improvement is pivotal, such as increased payload of UAVs, addition of active illumination to ground-based phenotyping platforms, automation of indoor phenotyping systems, implementation of FAIR data standards and new machine learning methods for analysis [11, 145–148]. Integration of the resulting phenotypes with other-omics data can enable a more comprehensive interpretation of sensor data and will eventually lead to a deeper understanding of plant-pathogen interactions.

## Data Availability

Data sharing not applicable to this article as no datasets were generated or analysed during the current study.

## Abbreviations

CCD: Charge-coupled device
Chl-F: Chlorophyll *a* fluorescence
CNN: Convolutional neural network
CT: Computed tomography
FAIR: Findable, accessible, interoperable, reusable
F_m_: Maximum fluorescence
F_v_/F_m_: Maximum quantum efficiency of PSII
IR: Infrared
LDT: Lesion doubling time
NIR: Near-infrared
NPQ: Non-photochemical quenching
OCT: Optical coherence tomography
PAM: Pulse-amplitude-modulated
PMMoV: Pepper mild mottle virus
PPI: Plant-pathogen interaction
PSII: Photosystem II
PSM: Plant secondary metabolite
qP: Photochemical quenching
RGB: Red–green–blue
SPCSV: Sweet potato chlorotic stunt virus
SPFMV: Sweet potato feathery mottle virus
SWIR: Shortwave-infrared
TIR: Thermal infrared
TMV: Tobacco mosaic virus
UAV: Uncrewed aerial vehicle
UV: Ultraviolet
UV-C: Ultraviolet C
VIS: Visible
